# Bacterial Tolerance to 1-Butanol and 2-Butanol: Quantitative Assessment and Transcriptomic Response

**DOI:** 10.3390/ijms252413336

**Published:** 2024-12-12

**Authors:** Alexander Arsov, Penka Petrova, Maria Gerginova, Lidia Tsigoriyna, Nadya Armenova, Ina Ignatova, Kaloyan Petrov

**Affiliations:** 1 Institute of Microbiology, Bulgarian Academy of Sciences, 1113 Sofia, Bulgaria; al.arsov@microbio.bas.bg (A.A.); ppetrova@microbio.bas.bg (P.P.); mariagg@microbio.bas.bg (M.G.); 2 Institute of Chemical Engineering, Bulgarian Academy of Sciences, 1113 Sofia, Bulgaria; lidinka29@gmail.com (L.T.); nadq.armenova@gmail.com (N.A.); ina.ignatova@iche.bas.bg (I.I.)

**Keywords:** 1-butanol, 2-butanol, butanol tolerance, *E. coli*, *Bacillus subtilis*, transcriptomics

## Abstract

The unique fuel characteristics of butanol and the possibility of its microbial production make it one of the most desirable environmentally friendly substitutes for petroleum fuels. However, the highly toxic nature of 1-butanol to the bacterial strains makes it unprofitable for commercial production. By comparison, 2-butanol has similar fuel qualities, and despite the difficulties in its microbial synthesis, it holds promise because it may be less toxic. This paper is the first comprehensive study to compare bacterial tolerance to different butanol isomers by examining the growth of 31 bacterial strains under 1-butanol and 2-butanol stress conditions. The presented results reveal that all tested strains showed a higher tolerance to 2-butanol than to 1-butanol at each solvent concentration (1%, 2%, and 3% *v*/*v*). Moreover, with an increased solvent concentration, bacterial cells lost their resistance to 1-butanol more rapidly than to 2-butanol. A comparison of the transcriptome profiles of the reference strains *Bacillus subtilis* ATCC 168 and *E. coli* ATCC 25922 disclosed a specific response to butanol stress. Most notably, in the presence of 2-butanol *E. coli* ATCC 25922 showed a reduced expression of genes for chaperones, efflux pumps, and the flagellar apparatus, as well as an enhancement of membrane and electron transport. *B. subtilis*, with 2-butanol, did not perform emergency sporulation or escape, as some global transcriptional stress response regulators were downregulated. The overexpression of ribosomal RNAs, pyrimidine biosynthesis genes, and DNA- and RNA-binding proteins such as *pcrA* and *tnpB* was crucial in the response.

## 1. Introduction

Fossil fuels meet 80% of the global energy need, and oil is expected to be the world’s most indispensable energy source by 2030 [1]. However, the American Petroleum Institute (API) has warned the United Nations that global oil production will likely cease between 2062 and 2094 due to the depletion of reserves. A reliable “green” alternative to petroleum derivatives is butanol, the fuel of the future [2], and it meets this need with its excellent fuel characteristics, low carbon emissions, and the possibility of biotechnological production. This is why the consumption of all butanol isomers has gradually increased over the last decade, and according to statistics, the 1-butanol market in 2023 reached USD 8 billion, whereas that of 2-butanol reached 10 billion, with a forecast of USD 17 billion by 2036. Butanol is an indispensable industrial solvent and platform chemical [3]. It is widely used in the chemical industry for methyl ethyl ketone production, in latex paints, varnishes, and plastics, and in cosmetics, pharmaceuticals, and medicine to synthesize drugs, vitamins, antibiotics, and hormones [4]. Butanol’s most important and promising use, however, is as fuel.

Compared to ethanol, which gained ground as a first-generation biofuel, a significant advantage of butanol lies in its full applicability to current spark-ignition engines due to its longer carbon chain. Butanol has a higher air–fuel ratio (11.1 vs. 9.0 for ethanol), a 25% higher energy density (29.2 vs. 19.6 MJ/L), lower hygroscopicity, lower vapor pressure and volatility, and excellent combustion characteristics [5,6]. It is also less corrosive, can be mixed with any concentration of gasoline, and can be transported through existing oil pipelines [7]. A comparison of the physicochemical features of 1-butanol and 2-butanol shows very similar fuel characteristics, indicating that the microbial production of 2-butanol may be economically viable (Table 1).

Biobutanol is obtained by the natural metabolism of anaerobic bacteria of the genus *Clostridium*, but also by genetically modified *Escherichia coli*, *Saccharomyces cerevisiae*, and algae that express synthetic butanol operons [9,10,11]. However, due to the toxicity of the final product, the titer and productivity achieved in batch fermentation are insufficient for commercialization. For example, around 20 g/L 1-butanol can be produced by *Clostridium* spp., with an average productivity of 0.5 g/L/h and a typical yield of 0.33 g/g substrate [9]. The highest 1-butanol concentration, which a recombinant *E. coli* obtained, is 20 g/L [10].

As an organic solvent, 1-butanol is known to attack the microbial cell membrane, intercalating the phospholipid bilayer and damaging lipopolysaccharides, thus causing cell content leakage [11,12,13]. It also induces severe changes in fatty acid synthesis, DNA damage, stress protein overexpression, efflux rearrangement, and membrane ATPase inhibition, ceasing glucose consumption [14,15,16,17]. However, there are no comprehensive data on whether the impact of 2-butanol is the same and on how bacterial cells respond to 2-butanol stress conditions. In our previous study of bacterial tolerance to 1-butanol, in which 74 strains of lactic acid bacteria were included, a connection between the hydrophobicity of the cell surface and the degree of tolerance was shown [18]. Other authors also reported the correlation between the hydrophobicity of various alcohols, esters, and carboxylic acids, including butanol, and the growth inhibition of *E. coli* [19,20]. The octanol/water partition coefficient (log P) reveals that the linear 1-butanol has higher hydrophobicity (logP 0.88) than isobutanol (0.80) and 2-butanol (0.61). The level of toxicity corresponds to the level of branching and hydrophobicity, as the current data reveal that branched butanol isomers are less toxic to *E. coli* DH5α than 1-butanol at all concentrations up to 15 g/L [21]. Studies in rats have shown that 2-butanol was about half as toxic four hours after it was inhaled [22].

Based on its physicochemical characteristics and notably lower hydrophobicity, 2-butanol may be less toxic to microbial strains than 1-butanol, although no such comparative studies exist. Transcriptomic investigations of 1-butanol tolerance showed that butanol affects multiple genes and their expression, but with several common mechanisms for Gram-positive and Gram-negative bacteria. First, butanol stress causes efflux pump overexpression, including of RND and ABC transporters of *E. coli* [23,24], SrpABC of *Pseudomonas putida* [25], and their analogs in *Clostridium* sp. [26]. Another effect is the upregulation of all stress-related proteins and chaperones: GroESL in *E. coli* [27]; GroESL in *C. acetobutylicum* ATCC 824 [28]; HtpG, GroEL, DnaK, HtrA, YacI, GroES, ClpP, Map, TufA, GreA, GrpE, and ClpC in *C. acetobutylicum* RH18 [29]; and DnaK, OmpJ, and CspA in *Ps. putida* BIRD1 [30]. Redox-related genes are usually turned off; membrane sugar transport is often impaired due to the downregulation of PTS transporters [31]. The most critical response, however, is the fine-tuning of global transcription regulators, as in Gram-negative bacteria, the overexpression of GntR leads to the activation of the oxidative stress cascade, an improvement in energy metabolism, and increased motility. In contrast, in Gram-positive bacteria, the enhancement of Spo0A expression leads to increased synthesis of all stress proteins, transcriptional arrest, and the targeting of sporulation [3].

Given the enormous toxicity of 1-butanol, scientists are currently focusing on developing biotechnology to obtain its supposedly less toxic structural isomer, 2-butanol. However, the production of 2-butanol in a biorefinery is also challenging, as only a few lactic acid bacteria can synthesize it from the substrate meso-2,3-butanediol. Russmeyer et al. [32] recently revealed that *Lentilactobacillus diolivorans* ATCC 14421 can convert meso-2,3-BD to 2-butanol through two consecutive enzymatic steps carried out by a diol dehydratase (encoded by *pduCDE* genes) and by alcohol dehydrogenase PduQ. Attempts to engineer *Klebsiella pneumoniae* to produce 2-butanol have also been performed [33]. After co-culturing engineered *Lactococcus lactis* and natural *Levilactobacillus brevis*, Mar et al. [34] achieved a 5.9 g/L 2-butanol titer with a high yield of 0.58 mol/mol glucose. Although the highest titer of 2-butanol obtained remains insufficient for industrial production, investigating 2-butanol toxicity levels is crucial for the development of 2-butanol production bioprocesses and strain engineering. Evidence that 2-butanol is less toxic than 1-butanol and an evaluation of the most resistant species would provide a clear guideline on the applicability of a particular bacterial species as a host for the 2-butanol metabolic pathway and on the limit of its productivity.

Therefore, this study aimed to broadly investigate bacterial tolerance to 2-butanol compared to 1-butanol, including Gram-positive and Gram-negative bacteria strains. Since these bacterial types show significant differences in their stress responses at the transcriptomic level, the second purpose of this study was to examine and compare the transcriptomic profiles of model strains (*E. coli* and *B. subtilis*) under stress conditions caused by the two butanol isomers.

## 2. Results

### 2.1. Bacterial Tolerance to 1-Butanol and 2-Butanol

All tested strains were cultured in media supplemented with 1-butanol or 2-butanol (1%, 2%, and 3% *v*/*v*) or without butanol (control). Bacterial growth was monitored by measuring cell density at OD_600_. All 31 strains (22 Gram-positive and 9 Gram-negative) from 25 species and 14 genera showed reduced growth in the presence of both butanol isomers (Table 2). Bacterial tolerance to both butanol isomers appeared to be genus- and strain-specific. All tested strains, regardless of genus and species affiliation, showed a lower reduction in specific growth rate in a medium containing 2-butanol compared to 1-butanol at each solvent concentration. Furthermore, with increasing solvent concentrations, cell tolerance to 1-butanol decreased much more significantly than that to 2-butanol. Thus, while all 31 strains grew at 2% 2-butanol, 12 strains (6 Gram-positive and 6 Gram-negative) did not grow at 2% 1-butanol. At the highest concentration tested, 3%, 6 of the Gram-positive and no Gram-negative strains showed any growth on 1-butanol, while 19 Gram (+) and 5 Gram (−) strains grew on 2-butanol.

**Figure 1:**
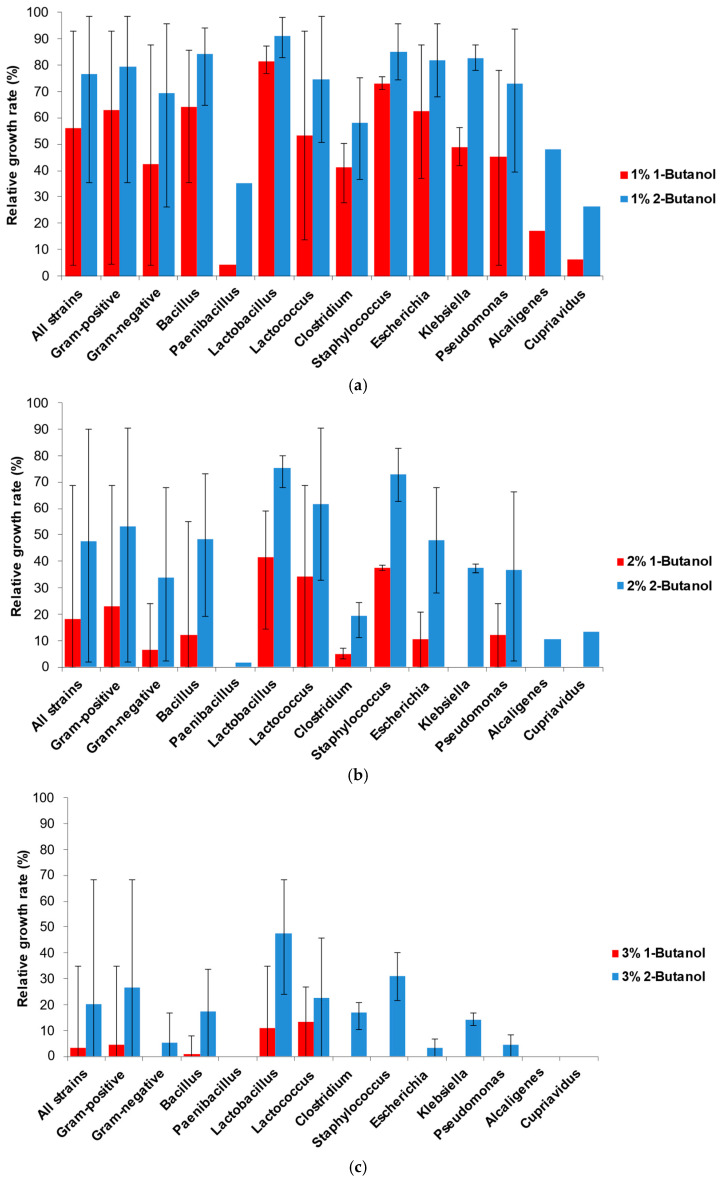
Relative growth rates of Gram-positive and Gram-negative genera in media with different concentrations of 1-butanol and 2-butanol. (**a**) 1% *v*/*v*; (**b**) 2% *v*/*v*; (**c**) 3% *v*/*v*.

Figure 1 shows the mean values for each bacterial genus tested for tolerance to both butanol isomers. The calculated average relative growth rate (RGR) of all tested bacterial strains was 76.49% at 1% 2-butanol, 56.24% at 1% 1-butanol, 47.48% at 2% 2-butanol, and 18.21% at 2% 1-butanol. At the highest concentration of 3%, the average RGR was 20.40% on 2-butanol and only 3.2% on 1-butanol. The representatives of some genera, such as the members of *Lactobacillus*, *Staphylococcus*, *Clostridium*, and *Klebsiella*, possessed similar relative growth rate values, revealing genus-specific tolerance. The deviation was ample in the genera *Pseudomonas*, *Escherichia*, and *Lactococcus*, revealing heterogeneity within each genus and strain-specific tolerance. Gram-positive bacteria appeared more resistant to butanol isomers, especially at 2 and 3% butanol concentrations. When 2% 2-butanol was added, the mean RGR of Gram-positive bacteria was 53.06%, versus that of Gram-negative bacteria, 33.83%, and at 3%, the rates were 26.55% versus 5.38%, respectively. At 3% 1-butanol, Gram (−) bacteria did not grow at all, while Gram (+) bacteria still had a mean RGR of 4.5%.

### 2.2. Transcriptomic Response to Butanol Stress of Reference Strains E. coli ATCC 25922 and B. subtilis ssp. subtilis ATCC 168

**Figure 2:**
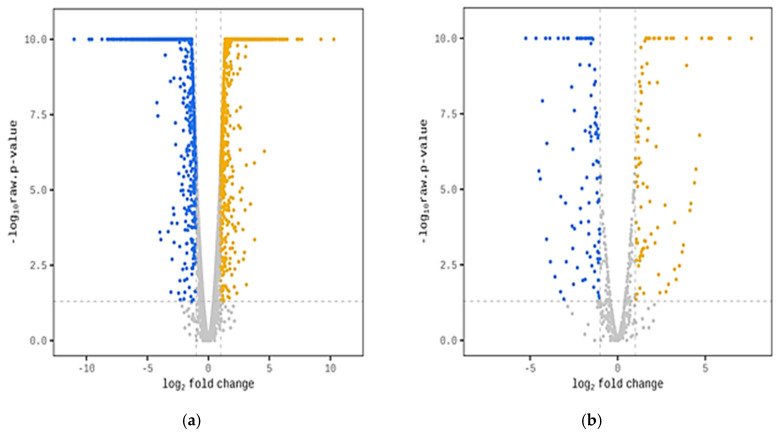
Volcano plots showing significant differentially expressed genes in the bacterial cells grown on 2-butanol vs. 1-butanol. Log2 fold change (FC) and *p*-value are presented. (**a**) *E. coli* ATCC 25922; (**b**) *B. subtilis* ATCC 168. Designations: yellow—FC ≥ 2; blue—FC ≤ (−2); raw *p*-value < 0.05.

The transcriptomic response to butanol stress caused by the two structural isomers was investigated using model species of Gram-positive (*B. subtilis* ATCC 168) and Gram-negative bacteria (*E. coli* ATCC 25922) to further elucidate the molecular mechanisms of butanol tolerance. The volcano plots for the two different species studied, shown in Figure 2, illustrate the scatter of the log10-transformed adjusted *p*-value versus the log fold change for each sample in the analysis.

The differences in gene expression were analyzed at the time point corresponding to the linear range of exponential growth (about 2.5 h for *E. coli* and ~3.5 h for *B. subtilis*). The effect of the solvents on *E. coli* was studied at a concentration of 2% (*v*/*v*). In *B. subtilis*, due to its lower tolerance, sufficient biomass for RNA isolation could not be accumulated at 2% butanol; therefore, it was tested at 1% (*v*/*v*) of the solvents. In the presence of 2% 1-butanol, *E. coli* ATCC 25922 cells began to disintegrate partially, and *B. subtilis* ATCC 168 showed obvious sporulation only after 8 h of challenge, as well as a visible change in cell shape and content (Figure S1). These changes were milder when the bacteria were cultured in a 2-butanol medium and resembled the control.

An overview of DEGs in the presence of butanol isomers is presented in Table 3.

In *E. coli*, a difference in gene expression affected 2161 genes in total: 1039 were overexpressed, and 1122 were downregulated.

In *B. subtilis*, 213 genes changed their expression levels: 102 were upregulated, 111 were downregulated, and 29 had a more than 10-fold altered expression. Most of these genes, with few exceptions, remained unchanged in *E. coli* (Table 4).

#### 2.2.1. DEGs Encoding Membrane Proteins and Transporters

**Figure 3:**
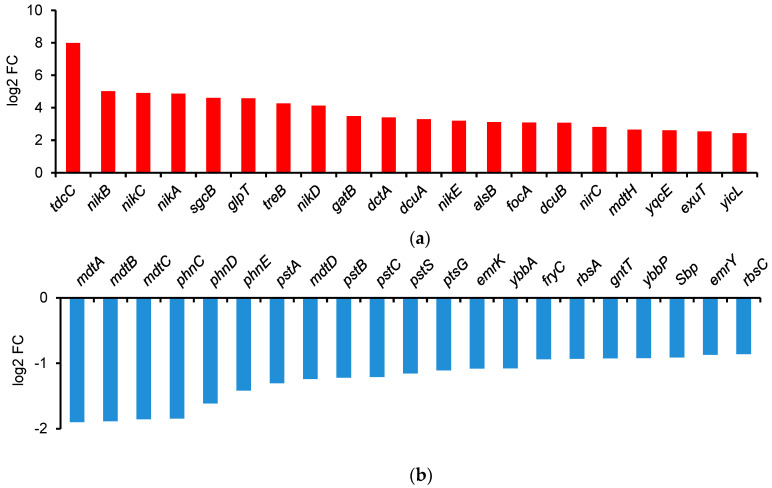
Differentially expressed genes (DEGs) in *E. coli* ATCC 25922, subjected to butanol stress (2% *v*/*v*). Fold change, as log2 FC, is estimated as the expression levels on 2-butanol vs. on 1-butanol. (**a**) Upregulated: *tdcC*—threonine/serine transporter; *nikB*, *nikC*, *nikA*—nickel ABC transporter permease subunits; *sgcB*—PTS sugar transporter IIB SgcB; *glpT*—glycerol-3-phosphate transporter; *treB*—PTS trehalose transporter IIBC; *nikD*—nickel import ATP-binding protein; *gatB*—PTS galactitol transporter IIB; *dctA*, *dcuA*, *dcuB*—C4-dicarboxylate transporters; *nikE*—nickel import ATP-binding protein; *alsB*—D-allose transporter; *focA*—formate transporter; *nirC*—nitrite transporter; *mdtH*—multidrug efflux MFS transporter MdtH; *yqcE*—MFS transporter; *exuT*—hexuronate transporter; *yicL*—carboxylate/amino acid/amine transporter. (**b**) Downregulated: *mdtA*, *mdtB*, *mdtC*, *mdtD*, *emrY*—multidrug efflux RND transporter permease subunits; *phnC*, *phnD*, *phone*—phosphonate ABC transporter proteins; *artJ*—arginine ABC transporter substrate-binding protein; *pstA*—phosphate permease; *pstB*, *pstC*, *pstS*—phosphate ABC transporter proteins; *ptsG*—PTS glucose transporter IIBC; *emrK*—multidrug efflux MFS transporter periplasmic adaptor; *ybbA*—ABC transporter ATP-binding protein—*Sbp*, sulfate/thiosulfate transporter; *rbsC*—ribose ABC transporter permease.

The proteome of the inner membrane of *E. coli* includes metabolite transporters, efflux pumps, and electron transport chains. Figure 3 shows the difference in gene expression on 2-butanol versus on 1-butanol.

All twelve of the most upregulated genes in *E. coli* ATCC 25922 were different types of transporters (Figure 3a). No fewer than 236 transporters were differentially expressed under 2-butanol compared to 1-butanol in *E. coli*, and this number corresponds to >10% of all regulated genes, indicating significant protein remodeling of the inner and outer membranes. Among the most significantly downregulated genes were the three subunits of the efflux pump MdtABC, all with 72 to 80 times lower expression (Figure 3b). Six other *mdt* genes (*mdtG*, *mdtG*, *mdtI*, *mdtJ*, *mdtN*, and *mdtQ*) were downregulated by between two and five times, while only two (*mdtM* and *mdtH*) were upregulated by four and six times, respectively. Ten other genes related to multidrug efflux transporters were found—six *acr* (A, B, D, E, F, R) and four *emr* (A, B, K, Y)—and all were downregulated by between 2 and 12 times. Among the most strongly upregulated genes were those for all subunits of the nickel transporter NikABCDE: *nikA* (29 times), *nikB* (32 times), *nikC* (30 times), *nikD* (17 times) and *nikE* (9 times); also upregulated—but only by 5 times—was *nikR*, the transcription repressor of *nikABCDE* which operates under Ni^2+^ excess.

**Figure 4:**
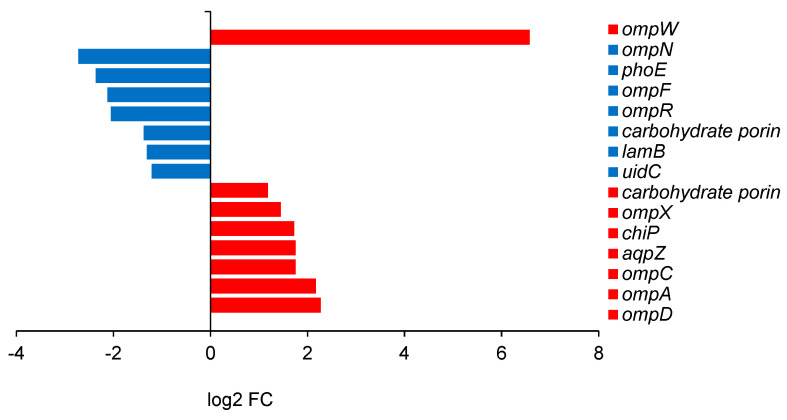
Differentially expressed genes encoding porins in *E. coli* ATCC 25922; log2 FC of 2-butanol versus 1-butanol is presented. Genes and proteins: *ompW*—outer membrane protein OmpW; *ompN*—porin OmpN; *phoE*—phosphoporin PhoE; *ompF*—porin OmpF; *ompR*—two-component system response regulator OmpR; *lamb*—maltoporin LamB; *uidC*—glucuronide uptake porin UidC; *ompX*—outer membrane protein OmpX; *chiP*—chitoporin; *aqpZ*—aquaporin Z; *ompC*—porin OmpC; *ompA*—porin OmpA; *ompD*—porin OmpD.

**Figure 5:**
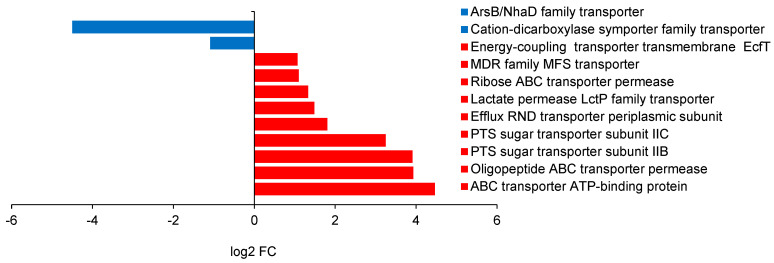
Differentially expressed genes for membrane transporters in *B. subtilis* ATCC 168; log2 FC applies to 2-butanol versus 1-butanol (1% *v*/*v*).

The outer membrane was also affected very differently by the different butanol isomers. Fourteen porins showed altered expression on 2-butanol (Figure 4), half upregulated and half downregulated, in both cases to a relatively small degree (2–7 times). Of the more important porins, *ompA* and *ompC* were upregulated by 4.5 and 3.4 times, respectively, while *ompF* was downregulated by 4.4 times. The most robust upregulation (95 times) was of *ompW*, one of the relatively little-known porins. *E. coli* phosphate and phosphonate transporters, encoded by *pst* and *phn* genes, respectively, were substantially reduced on 2-butanol, for instance, *phnC* (70 times), *phnD* (41 times), and *phnE* (26 times), and to a lesser extent, *pstA* (20 times), *pstB* (17 times), *pstC* (16 times), and *pstS* (14 times). On the other hand, among the most significantly upregulated genes were those for PTS transporters for trehalose (*treB*—19 times) and galactitol (*gatB* and *gatC*—both 11 times); two C-4 dicarboxylate transporters (*dctA* and *dcuA*—both 10 times), and one for glycerol-3-phosphate (*glpT*—24 times). The pattern in *B. subtilis* was essentially different (Figure 5), with fewer and different genes affected, with most of them (9 out of 11) upregulated by between 2 and 22 times.

#### 2.2.2. DEGs Encoding Proteins of Flagella, Sporulation, and Electron Transport Chains

Gene clusters responsible for the flagellar apparatus and the electron transport chains in *E. coli* were affected in different directions. Of 20 genes related to flagellum and organized into three clusters (*flg*, *flh*, and *fli*), plus *flk*, which encodes a biosynthesis regulator, 19 were downregulated by between two and five times. This is consistent with the upregulation by 2.5 times of the *flhC* gene encoding the FlhC repressor. In *B. subtilis*, the “toxicant escape” gene encoding the flagellar motor switch protein FliM was 2.5-fold downregulated on 2-butanol. The gene encoding stage V sporulation protein SpoVS [35] was six-fold downregulated, consistent with the five-fold reduced expression of the RsfA family sporulation regulator gene, both testifying to the conclusion that sporulation was unnecessary under 2-butanol stress. The electron transport chains in *E. coli* were consistently strengthened, especially the five genes (A–E) from the *cyo* operon (four subunits of the cytochrome oxidase and one gene for a heme synthase), which were upregulated by 11 to 16 times.

Sixteen genes (A–C, E–L) from the *nuo* operon, which encodes the various subunits of the NADH–quinone oxidoreductase complex, were consistently upregulated by between three and seven times.

#### 2.2.3. DEGs Encoding Transcriptional and Global Regulators

The number of differentially expressed transcription factors in *E. coli* was 128, and in *B. subtilis*, it was 14. Table 5 summarizes the most important of them.

In *E. coli*, the expression of several important stress response regulators was reduced—those for acid stress (YdeO), phosphate limitation (PhoB), and superoxide stress (SoxS)—indicating a “low degree of alert” to 2-butanol as a solvent. This was also observed in *B. subtilis*; here, the Spx factor should be mentioned. This is a global transcriptional regulator of the oxidative stress response in Gram-positive bacteria that participates in the control of organosulfur utilization operons, including the *ytmI*, *yxeI*, *ssu*, and *yrrT* operons. Spx was almost 3-fold downregulated in *B. subtilis* 168 on 2-butanol compared to on 1-butanol. Also notable was the downregulation of Rrf2 (log2 FC −4.06, or 16.7 times), the master repressor of cysteine metabolism.

However, in *E. coli*, the genes for the transcriptional antiterminator BglG and the transcriptional activator NorR were significantly downregulated (log2 FC −6.64 and −6.11) to prevent the synthesis of unnecessary proteins under stress conditions.

**Figure 6:**
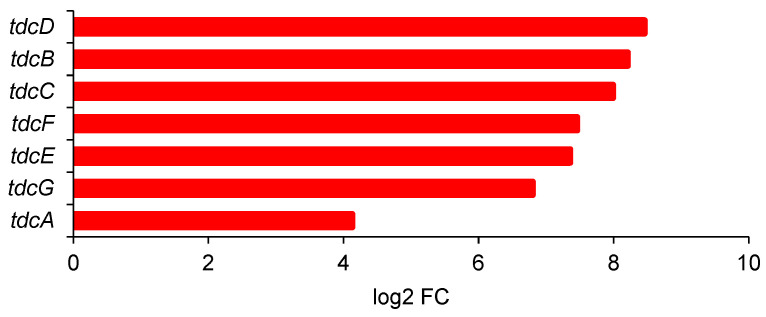
DEGs of *tdcABCDE* operon of *E. coli* ATCC 25922; log2 FC applies to 2-butanol versus 1-butanol (2% *v*/*v*).

Some transcription factors regulate the function of entire operons. Among the most strongly upregulated in *E. coli* was TdcA (17.5 times), the activator of the *tdcABCDE* operon. The other four genes of the operon were among the most strongly upregulated (Figure 6), from 164 to 350 times (log2 FC between 4.1 and 8.5), which suggests high levels of threonine degradation under butanol stress.

**Figure 7:**
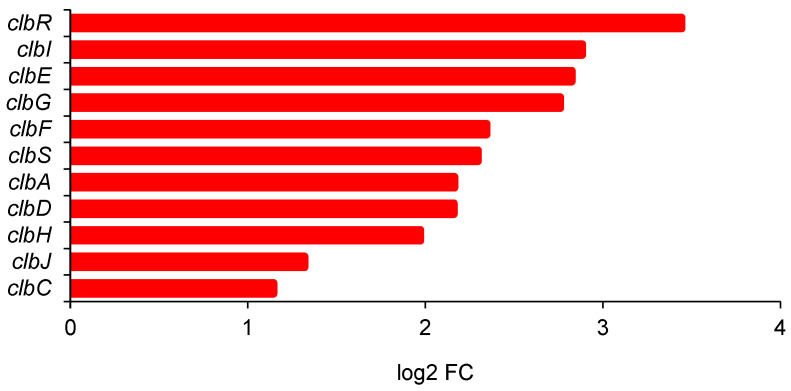
Colibactin biosynthesis gene cluster in *E. coli* ATCC 25922. Fold change of differentially expressed genes with 2-butanol versus 1-butanol (2% *v*/*v*). The genes encode the following proteins: *clbR*—LuxR family transcriptional regulator; *clbC*, *clbI*—polyketide synthases; *clbJ*, *clbH*—peptide synthetases; *clbD*, *clbF*—dehydrogenases; *clbA*—transferase; *clbS*—self-protection protein; *clbG*—acyltransferase; *clbE*—amino malonyl-acyl carrier.

The transcription regulator ClbR, upregulated 11 times, is part of the *clp* gene cluster responsible for the biosynthesis of colibactin. Another 10 of its 19 genes were upregulated between two and seven times, corresponding to log2 FC values between 1.8 and 2.9 (Figure 7).

#### 2.2.4. DEGs Encoding Chaperones

A total of 32 chaperones were differentially expressed in *E. coli*, with 24 downregulated and 8 upregulated. The most strongly downregulated genes (*spy*, *ibpA*, *ibpB*) are involved in protein folding and refolding, while the most strongly upregulated gene (*hypA*) is involved in the maturation of Ni–Fe hydrogenases. Three chaperones were regulated in *B. subtilis*; all of them had increased expression by between 3.5 and 7.0 times (Table 6), and all of them, significantly, were downregulated in *E. coli* (from 2.2 to 3.5 times).

The synthesis of the ATP-independent periplasmic protein-refolding chaperone Spy was minimal (−4.86 log2 FC). These proteins are usually considered holdases that bind proteins and prevent their aggregation [36]. Still, under conditions of 2-butanol stress, it can be assumed that they are not needed in large quantities. The RNA chaperone *cspE*, one of the so-called ‘cold shock’ proteins, was upregulated by 23 times in *E. coli*; however, genes encoding proteins with similar functions in *B. subtilis* (CspB and CspD) were downregulated (Table 6).

#### 2.2.5. Other DEGs

**Figure 8:**
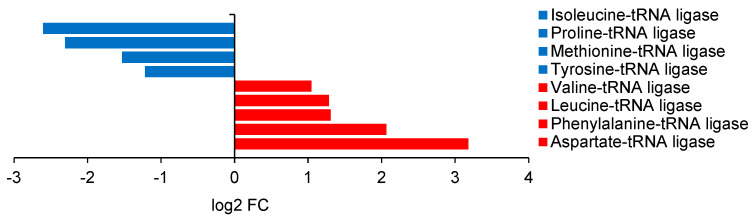
Regulated genes for tRNA ligases in *B. subtilis* ATCC 168.

In *B. subtilis*, two ribosomal RNA genes, 16S and 23S, were among the most strongly upregulated by 85 and 82 times, respectively (log2 FC > 6). However, this effect was not replicated in regard to ribosomal proteins. Four of these (L28, L31, L7Ae, and S1), plus one ribosomal-processing cysteine protease, were consistently downregulated by between two and five times. A similar discrepancy was observed among various tRNA ligases (Figure 8).

Five genes were upregulated between two and nine times (most strongly the tryptophan—tRNA ligase), and four were downregulated by between two and six times (most strongly the isoleucine—tRNA ligase). However, not one tRNA gene was found to be regulated in either direction.

Two of the most pronounced upregulations were of genes involved in pyrimidine synthesis in *B. subtilis*: *pyrF*, or orotidine-5′-phosphate decarboxylase—40 times; and *carB*, or the large subunit of carbamoyl-phosphate synthase—39 times. Two subunits from dihydroorotate oxidase, *pyrD*, and *pyrK*, were upregulated by 3.3 and 2.4 times, respectively. However, no significant changes were observed in the expression of the remaining genes for enzymes of this metabolic pathway.

## 3. Discussion

Since in most cases, bacterial strains produce butanol in amounts approximately equal to those they tolerate [37], the significance of the microbial production of 2-butanol is in the presumed greater tolerance to it compared to 1-butanol. The effect of 1-butanol was most thoroughly studied in *C. acetobutylicum* ATCC 824, revealing that its growth was inhibited by 50% at a concentration of 0.9% (*w*/*v*) and inhibited completely at 1.6% (*w*/*v*) [38]. *E. coli*, even recombinant strains designed for increased butanol tolerance, do not tolerate more than 1.5–2.0% butanol [3]. A naturally high tolerance to 1-butanol has been reported by Kanno et al. [39] for representatives of the genera *Bacillus*, *Brevibacillus*, *Lysinibacillus*, *Rummeliibacillus*, *Caloribacterium*, *Coprothermobacter*, and *Enterococcus*. The whole group of lactic acid bacteria possessed the same property, with the highest resistance to 1-butanol (up to 4% *v*/*v*) found in *Pd. acidilactici* and *Lp. plantarum* [18,34]. Many naturally butanol-resistant strains have been used to obtain mutants or recombinants with higher levels of 1-butanol production [3,33].

As concerns 2-butanol, some natural producers highly tolerate it. For example, *Lent. diolivorans* LMG 19667 tolerates 2-butanol at a concentration of 2.5% and reaches 88% of the maximum OD_600_ after 72 h of incubation [32]. *K. pneumoniae* HR526 tested by Chen et al. showed tolerance to 3% of both 1-butanol and 2-butanol for 24 h. The estimation of the final OD_600_ showed a 63.3% reduction in the presence of 1-butanol and only 18.9% in the presence of 2-butanol [33]. For *Lc. lactis*, at the same concentration of 3% of 2-butanol, a decrease in the specific growth rate of 60% was reported, while at 2.5%, *Levilactobacillus brevis* strain SE20 decreased its growth by 49% [40].

The effect of 1-butanol, which is more hydrophobic and has been proven toxic to microorganisms, is extensively documented. All isomers of butanol disrupt the lipid bilayer of the microbial cell. The interaction between alcohols and membranes is highly complex and likely to affect a wide range of its properties, probably including membrane proteins [41]. In vitro and silico studies suggest that butanol attacks mostly with the polar headgroups at the outer edge of the lipid bilayer, yet also causes partial intercalation and increased disorder among the acyl chains [42]. Lipidomic studies of *C. saccharoperbutylacetonicum* N1-4 show significant changes in their lipid profiles under butanol stress [43].

However, little is known about the overall effects of 2-butanol on microbial cells. One of the few studies of this topic is on recombinant *E. coli* containing and overexpressing the autologous GroESL chaperone system, which has been shown to increase the strain’s butanol tolerance. Both the control and the recombinants were found to be less tolerant to 1-butanol and isobutanol (1% both) than to 2-butanol (1.25%) after 24–48 h of cultivation [11].

**Figure 9:**
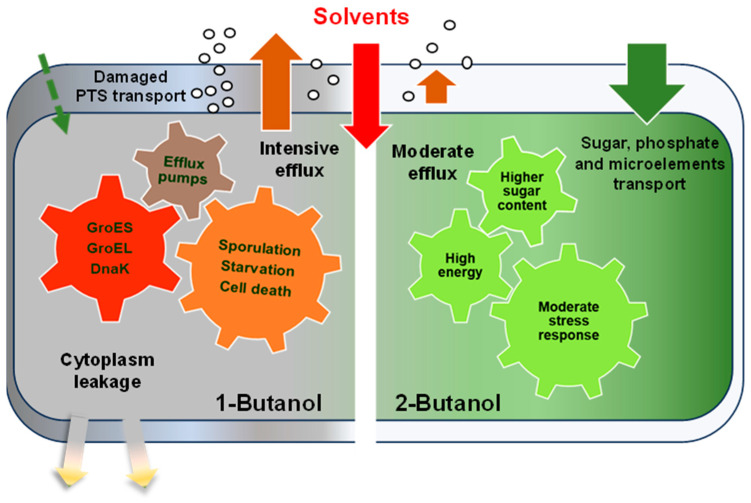
A comparative scheme of the effects of 1-butanol and 2-butanol on the microbial cell, as revealed by transcriptomic results.

The present investigation is the first serious effort to evaluate the toxicity of 2-butanol to bacterial cells and compare it with that of 1-butanol (Figure 9).

The lower toxicity of 2-butanol, theoretically predicted based on its lower hydrophobicity, has been experimentally verified for 31 strains from 25 species and 14 genera. All investigated strains demonstrated a higher tolerance to 2-butanol than to 1-butanol, with increased differences at higher solvent concentrations. As expected, Gram-positive bacteria, shielded by a thick peptidoglycan layer, displayed higher butanol tolerance than Gram-negative bacteria. Thus, all tested Gram (+) strains grew on 2% 2-butanol, while 19 of them grew on 3% 2-butanol.

Transcriptomics has been used surprisingly little so far in the context of butanol stress. One example of its successful application includes cyanobacteria (*Synechocystis* PCC 6803) so sensitive to butanol that even 1 g/L was enough to cause a difference in the expression of 280 genes. Six of these genes, mostly concerned with electron transport and oxidative stress, were chosen for overexpression, which increased both the growth rate and the viability of cyanobacteria on 4 g/L butanol, even under conditions of severe butanol shock (20 g/L) [44]. Similar studies on *E. coli* are less impressive, yet the overexpression of several genes produced 10–35% increased survival. The single most potent effect was achieved by the overexpression of *ygfO*, encoding a proton motive xanthine transporter [45]. A recent comparative transcriptomic study of butanol-tolerant and butanol-resistant strains of *Lp. plantarum* revealed that 1-butanol (2% *v*/*v*) exerted a massive effect on transcription. Altogether, 835 genes were differentially expressed: 104 in the same direction in both strains, indicating common stress mechanisms, 15 in opposite directions, and 716 uniquely in either of the two strains, including genes involved in membrane transport, tryptophan synthesis, and glycerol metabolism [31].

The present study is the first to examine differences in the transcriptional response of bacterial cells subjected to butanol stress by comparing gene expression upon 1-butanol and 2-butanol challenge. *E. coli* ATCC 25922 showed remarkably different transcriptome profiles, revealing a complex response mechanism to 1-butanol and 2-butanol, well beyond their hydrophobicity. Indeed, the greatest differences were in genes related to membrane transport. These are the porins and transport proteins, the efflux pumps, and the genes related to flagella. A downregulation of the genes encoding subunits of the efflux system was observed in the presence of 2-butanol compared to 1-butanol, for example, *mdtA*, *mdtB*, *mdtC*, *mdtD,* and *emrY*, multidrug efflux RND transporter permease subunits; *emrK*, etc. (Figure 2). This is an indication that 2-butanol is less toxic to the cell than 1-butanol and that its excretion outside the cell is not as urgent. In addition, genes for ABC transporters such as *pstA*, *pstB*, *pstC*, *ptsG*, and *pstS* (phosphate permeases), PTS glucose transporter IIBC, and *ybbA*, the ABC transporter ATP-binding protein, were also downregulated, revealing less energy starvation under 2-butanol stress conditions compared to under 1-butanol stress. In *B. subtilis*, some differences were observed. Still, the trend persisted, as the Asp23 family envelope stress response protein was downregulated two-fold in 2-butanol compared to in 1-butanol, and the ArsB family transporter gene (involved in various detoxifications) was downregulated 25-fold.

A recent proteomic study of *E. coli* subjected to short-chain alcohol stress, including 1-butanol, found increased levels of inner membrane transporters for the uptake of energy-producing metabolites, and reduced levels of many outer membrane β-barrel proteins (LptD, FadL, LamB, TolC, and BamA) and non-essential proteins related to different types of stress [46]. The last effect is to some extent mirrored in our data by the reduction in the efflux pumps and the transporters for phosphates and phosphonates; the first effect may be said to be analogous to the upregulation of transporters for trehalose, galactitol, and glycerol-3-phosphate. However, we observed different effects with genes encoding distinct proteins in terms of porins. Porins are trimeric β-barrel proteins that form passive channels for low-molecular-weight substrates in the outer membrane of *E. coli* [47]. OmpC and OmpF are the most important for transportation, while OmpA plays a vital role in stabilizing the outer membrane by interacting with the peptidoglycan layer in the periplasmic space [48]. OmpC is notably reduced by alcohol stress, and the outer membrane is destabilized. Increased membrane permeability destroys the proton gradient and disrupts ATP synthesis, which may cause an energy shortage. In this view, the upregulation of *ompC* and *ompA* we observed, while relatively small, could be substantial. Interestingly, however, *ompF* was downregulated. Further studies are necessary, including extensive lipidomic analysis, to completely elucidate the role of the outer membrane in Gram-negative bacteria under butanol stress.

One of the most surprising effects we observed was the enormous upregulation of *ompW*. OmpW appears to be an ordinary porin, a small eight-stranded β-barrel protein that forms a hydrophobic channel in the outer membrane [49]. It has been connected with resistance to phagocytosis [50], cationic influx together with the small multidrug resistance (SMR) protein EmrE [51], the transition from an aerobic to anaerobic lifestyle, and susceptibility to colicin [52]. The upregulation of *ompW* has also been connected with butanol tolerance [53], but never to such an extent and never concerning 2-butanol.

The MdtABC efflux system is involved in the disposal of several toxic compounds, such as novobiocin, bile salts, detergents, and zinc. Still, its action on alcohol has not been reported so far [54]. A considerable reduction in BglG and NorR is likely to reduce energy expenditure for the performance of less urgent functions, as BglG exerts positive regulation of the bgl (beta-glucoside) operon, conferring the ability to use β-glucosides as carbon sources [55]. A reduction in NorR, among the first transcriptional activators of the NO reductase to be discovered, would make the microbial cell more susceptible to the threat of nitric oxide, but it seems that under butanol stress, this is a risk worth taking [56].

The TdcABCDE operon has been studied intensively since the late 1980s [57,58]. It is involved in the transport and degradation of threonine, and to a lesser extent, also of serine, mostly during anaerobic growth [59]. Our data suggest that it is strongly involved in butanol stress, and its upregulation may be one of the key factors in the lower toxicity of 2-butanol. Significantly, the most substantial upregulation (350 times) was of the gene encoding TdcD, propionate kinase, which catalyzes the conversion of propionyl phosphate to propionate, the only step in which ATP is generated.

The effect we observed on the NikABCDE transporter is among the strongest and most consistent of all. Despite some attempts to lump the nickel transporter in *E. coli* together with other ABC transporters into some families [60], the substrate-binding subunit NikA is highly specific to nickel. Even Co^2+^ ions are bound with 20 times lower affinity, and Ca^2+^ ions are not bound at all [61]. Nickel homeostasis in *E. coli* is critical under anaerobic conditions due to the expression of Ni–Fe hydrogenase isozymes, the subject of intricate regulation. At sufficiently low concentrations, nickel promotes biofilm formation in *E. coli* [62]. It may be one of the key factors in the bacterial response to butanol stress as well, but further studies are needed to establish this. The biosynthesis of colibactin, a genotoxic metabolite with a potentially cancerogenic effect (a polyketide peptide with DNA crosslinking ability), appears to be of lesser importance, yet 10 of the 19 genes in the *clb* gene cluster (*clb*A to *clb*S) [63] appear to be consistently upregulated.

The transcriptomic response in *B. subtilis* 168 revealed profound differences from that in *E. coli* ATCC 25922, suggesting entirely different mechanisms for coping with butanol stress in Gram-positive and Gram-negative bacteria. One genomic and transcriptomic study of *Lc. lactis* obtained via adaptive laboratory evolution (ALE) and challenged with 40 g/L isobutanol found that the most significant instances of upregulation affected amino acid metabolism and membrane transport, while the most downregulated genes were involved in translation and carbohydrate metabolism [64]. Our data agree, up to a point, but only in regard to the membrane transporters, of which 9 out of 11 were upregulated. The sporadic upregulation of enzymes involved in pyrimidine biosynthesis is a curious effect that needs further study to ascertain its importance. Perhaps it is worth noting that only one of the four genes (*carB*) was also regulated in *E. coli* ATCC 25922, in the opposite direction (−2.09). Interestingly, an earlier study found an inhibition of pyrimidine biosynthesis in the butanol-resistant *L. plantarum* Ym-1 subjected to 2% butanol. In this case, however, the effect was much more pronounced and consistent [31].

The most significant upregulation in *B. subtilis* 168, by 197 times, was of the gene *tnpB*. TnB is an RNA-guided DNA nuclease, an evolutionary predecessor of CRISPR-related nucleases such as Cas9 and Cas12 [65]. It has no analog in the transcriptomic response of *E. coli* ATCC 25922, and neither does the massive upregulation of 16S and 23S ribosomal RNAs. However, this effect should be confirmed by the upregulation of at least some of the 86 tRNAs in the genome of *B. subtilis* [65], preferably in combination with some genes for ribosomal proteins, before some validity is ascribed to it.

There was very little overlap in the transcriptomic responses of *B. subtilis* 168 and *E. coli* ATCC 25922. The gene *pcrA* in *B. subtilis*, encoding a helicase involved in rolling-circle replication and repair [66], was upregulated by 18 times, while *dnaB* and *dnaC*, two of the helicases in *E. coli*, were downregulated by 2 and 3.5 times, respectively; *uvrD*, the most abundant helicase in *E. coli*, was not regulated at all. Cold shock proteins, RNA chaperones involved in post-transcriptional regulation, showed something like a pattern. Only three of them were regulated in *B. subtilis*, namely, *cspB*, *cspD*, and one unidentified protein, all reduced in expression, while in *E. coli*, no fewer than eight were differentially expressed, with five of them downregulated from 3 to 23 times (*cspG*, *cspH*, *cspI*, *ymcF*, *ynfQ*) and three upregulated to the same degree (*cspD*, *cspE*, *ydfK*). Future research will establish whether these areas of mild overlapping or unique regulation are the keys to the vastly different effects of butanol in Gram-positive and Gram-negative bacteria.

## 4. Materials and Methods

### 4.1. Bacterial Strains, Media, and Cultivation Conditions

The bacterial strains used in this study are listed in Table 2. They were obtained from the microbial collections ATCC (American Type Culture Collection, Manassas, VA, USA), DSMZ (German Collection of Microorganisms and Cell Cultures GmbH, Braunschweig, Germany), the Bulgarian National Bank for Industrial Microorganisms and Cell Cultures (NBIMCC, Sofia, Bulgaria), or the microbial collection of the Institute of Microbiology, Bulgarian Academy of Sciences.

A Luria–Bertani (LB) medium was used to grow and analyze all *Bacillus* spp., *Paenibacillus polymyxa*, *E. coli*, *S. aureus*, *A. faecalis*, *K. pneumoniae*, *C. necator*, and *Pseudomonas* spp. All representatives of lactic acid bacteria (lactobacilli and lactococci) were grown in MRS, while *Clostridium* spp. were analyzed using DSMZ medium 104 with a pH of 7.0, containing the following (g/L): trypticase peptone—5.0; pepsin-digested meat peptone—5.0; yeast extract—10.0; L-cysteine—0.5; Na_2_CO_3_—1.0; D-glucose—5.0; sodium resazurin—0.1% (*w*/*v*); and salt solution—40.00 mL (CaCl_2_ × 2H_2_O, 0.25; MgSO_4_ × 7H_2_O 0.50; K_2_HPO_4_, 1.0; KH_2_PO_4_, 1.0; NaHCO_3_, 10.0; NaCl, 2.0).

The strains were cultured in 500 mL flasks containing a 100 mL medium on a rotary shaker under aerobic conditions (New Brunswick, NJ, USA).

*Clostridium* spp. strains were cultivated in 100 mL bottles under anaerobic conditions, in a preliminary prepared anoxic medium (sparged with 80% N_2_ and 20% CO_2_ gas mixture for 30 min).

All strains were cultivated at their optimal temperatures, ranging between 30 °C and 37 °C.

### 4.2. Butanol Tolerance Assay

Butanol tolerance was estimated by cultivating each strain in appropriate broth containing 1-butanol or 2-butanol (Sigma-Aldrich Chemie GmbH, Schnelldorf, Germany) in 1%, 2%, and 3% (*v*/*v*) concentrations. The inoculum had a concentration of 1% *v*/*v* and used overnight cultures grown in a 50 mL medium to approximately OD_600_ = 2.0.

To assess the degree of butanol resistance of each strain, the relative growth rate (RGR) in the medium supplemented with 1-butanol or 2-butanol was determined. The RGR was calculated as the ratio of the specific growth rates in the presence of butanol to the specific growth rates of the control (mediums without butanol) × 100 (%).

The specific growth rate (SGR) was estimated by the formula

SGR = (ln OD_600_ (t_2_) − ln OD_600_ (t_1_))/(t_2_ − t_1_)

### 4.3. Analytical Methods

Cell growth was monitored by measuring the optical density (OD) at wavelength λ = 600 using a UV/VIS Spectrophotometer (Thermo Scientific Inc., Waltham, MA, USA).

RNA concentrations and purity (Abs_260_/Abs_280_ ratio) were determined using the Quawell UV Spectrophotometer Q3000 (Quawell Technology, Inc., San Jose, CA, USA).

Microscopic observations were conducted using a Leika microscope (Wetzlar, Germany) with ×1000 magnification. A digital camera was used to document the images.

### 4.4. RNA Isolation, Library Construction, Next-Generation Sequencing, and Determination of Differentially Expressed Genes (DEGs)

Total RNA was isolated with the GENE Matrix Universal RNA Purification kit (EURx, Gdansk, Poland) according to the manufacturer’s protocol.

The cultures were grown for ~2.5 (*E. coli* ATCC 25922) or ~3.5 h (*B. subtilis* ATCC 168) in LB media with and without solvents.

Macrogen Inc. (Seoul, Republic of Korea) constructed the libraries and accomplished the sequencing. RNA-seq libraries were prepared from 1 µg of total RNA using TruSeq Stranded with the NEB rRNA depletion kit (Illumina, San Diego, CA, USA).

The copy-DNA synthesis and adaptor additions were performed according to Illumina protocol after RiboZero rRNA removal. After library quantification, 100 bp paired-end sequencing was conducted on Illumina HiSeq 2000. The raw paired-end reads were trimmed and quality-controlled using Trimmomatic v.0.38. Trimmed reads were mapped to the reference genome with Bowtie 1.1.2. The reads were separately aligned to the reference genomes of *E. coli* ATCC 25922 (GCF_000401755.1) and *B. subtilis* ssp. *subtilis* 168 (GCF_000009045.1).

To identify differentially expressed genes (DEGs), the expression levels of the transcripts were calculated using the fragments per kilobase of reading per million mapped reads (RPKM) method. The expression profile was calculated for each sample and gene as a reading count. The fold change (FC), ExactTest (using edgeR), and hierarchical clustering were used as statistical methods.

The transcriptomic data were deposited in the GenBank of NCBI into the SRA database under BioProjects accession numbers PRJNA1178085 (*E. coli* ATCC 25922) and PRJNA1178164 (*B. subtilis* ATCC 168).

## 5. Conclusions

This comparative study of bacterial tolerance to two structural isomers of butanol showed that all tested strains were more tolerant to 2-butanol compared to 1-butanol, with the differences being more prominent with increases in concentration (from 1% to 3% *v*/*v*). The transcriptomic profiling of reference strains *E. coli* ATCC 25922 and *B. subtilis* ATCC 168 revealed that 2-butanol induced weaker changes in gene expression than 1-butanol. This included less activation of genes encoding chaperones involved in damaged protein refolding and efflux pumps. In *E. coli*, the synthesis of membrane transport and electron transport chain proteins was upregulated. In *B. subtilis*, sporulation was overcome by the downregulation of responsible genes, and in both types of bacteria, the expression of genes encoding flagellar proteins was reduced.

Although industrial production of 2-butanol is still far from being realized, and only engineered strains can be employed, the presented results suggest that the microbial production of 2-butanol has at least one advantage. The observation that 2-butanol is less toxic to bacterial cells opens up the possibility of producing it in higher concentrations than 1-butanol and could be helpful in future investigations.

## Figures and Tables

**Table 1:** Comparison of the fuel properties of 1-butanol and 2-butanol.

Properties	1-Butanol	2-Butanol
Molar mass	74.123 g/mol	74.123 g/mol
Density ^1^	0.810 g/cm^3^	0.806 g/cm^3^
Melting point	−89.8 °C	−114.7 °C
Boiling point	117.7 °C	99.5 °C
Log P	0.88	0.61
Vapor pressure ^2^	0.58 kPa	1.67 kPa
Oxygen (wt %)	21.62	21.62
Energy content	33.19 (MJ/kg)	32.74 (MJ/kg)
Heating value	−2670 kJ/mol	−2661 kJ/mol
Autoignition temperature	343 °C	405 °C
Octane number	96	101
LC_LO_ ^3^	8000 ppm (rat, 4 h)	16,000 ppm (rat, 4 h)

^1^ At 25 °C; ^2^ at 20 °C; ^3^ LC_LO_—lethal concentration, the lowest published [8].

**Table 2:** The relative growth rate of bacterial strains in the presence of 1-butanol or 2-butanol compared to a control without butanol. The presented results are mean values of triplicate trials. RGR is the relative growth rate.

Species	Strain	RGR on 1-Butanol (*v*/*v*)			RGR on 2-Butanol (*v*/*v*)		
		1%	2%	3%	1%	2%	3%
**Gram-positive bacteria**							
*Bacillus licheniformis*	24	57.1 ± 2.8	11.2 ± 1.1	0	74.5 ± 4.3	48.0 ± 3.2	27.5 ± 4.0
*Bacillus licheniformis*	55	61.8 ± 3.3	3.5 ± 0.6	0	92.7 ± 3.4	43.7 ± 1.8	19.7 ± 2.9
*Bacillus subtilis*	ATCC 168	72.6 ± 3.4	0	0	90.7 ± 1.4	57.8 ± 3.3	33.8 ± 6.1
*Bacillus subtilis*	35	72.1 ± 5.4	0	0	91.4 ± 2.7	39.7 ± 2.1	9.7 ± 2.2
*Bacillus safensis*	14-A	56.7 ± 4.5	0	0	71.3 ± 5.5	42.6 ± 2.2	5.7 ± 2.8
*Bacillus velezensis*	5RB	73.0 ± 4.7	55.0 ± 6.3	8.0 ± 2.3	94.3 ± 2.4	73.3 ± 1.6	25.6 ± 3.1
*Bacillus thuringiensis*	BTG	85.7 ± 3.8	28.4 ± 4.0	0	93.6 ± 3.1	61.9 ± 4.4	18.7 ± 5.4
*Bacillus globiformis*	NBIMCC 2212	35.3 ± 2.2	0	0	64.6 ± 1.3	19.1 ± 0.8	0
*Paenibacillus polymixa*	NBIMCC 1126	4.4 ± 0.4	0	0	35.3 ± 2.1	1.9 ± 0.6	0
*Lacticaseibacillus paracasei*	DSM 23505	84.2 ± 5.2	29.7 ± 2.6	0	98.1 ± 1.7	75.0 ± 4.6	50.3 ± 4.8
*Limosilactobacillus fermentum*	ATCC 14932	87.4 ± 6.1	53.2 ± 7.3	13.9 ± 2.4	92.3 ± 1.4	79.2 ± 1.9	68.2 ± 3.7
*Lactiplantibacillus plantarum*	NCDO 1193	78.4 ± 4.2	35.3 ± 2.5	4.5 ± 2.0	92.4 ± 3.0	71.9 ± 2.4	40.6 ± 6.5
*Lacticaseibacillus rhamnosus*	ATCC 7469	77.9 ± 5.3	14.5 ± 0.5	0	91.8 ± 3.3	68.0 ± 6.2	24.2 ± 3.9
*Lacticaseibacillus casei*	ATCC 27139	76.9 ± 3.2	58.9 ± 5.0	11.7 ± 2.8	82.8 ± 0.9	79.9 ± 2.2	38.9 ± 4.3
*Lentilactobacillus diolivorans*	DSM 14421	83.4 ± 4.3	58.9 ± 1.6	34.8 ± 4.5	88.7 ± 7.0	78.0 ± 3.9	62.3 ± 3.1
*Lactococcus lactis*	DSM 20481	92.8 ± 3.7	68.7 ± 4.0	26.7 ± 5.2	98.6 ± 2.6	90.5 ± 2.0	45.7 ± 5.8
*Lactococcus lactis*	IL 1403	13.6 ± 1.7	0	0	50.6 ± 5.2	32.8 ± 3.5	0
*Staphylococcus aureus*	ATCC 29213	70.6 ± 4.8	36.7 ± 3.2	0	74.2 ± 6.2	62.8 ± 5.7	21.5 ± 4.1
*Staphylococcus aureus*	ATCC 23235	75.4 ± 5.0	38.5 ± 4.1	0	95.7 ± 3.4	82.8 ± 5.2	40.3 ± 5.6
*Clostridium acetobutylicum*	DSM 792	45.3 ± 3.1	7.3 ± 0.5	0	62.8 ± 5.3	24.6 ± 2.1	20.1 ± 3.6
*Clostridium beijerinckii*	DSM 51	27.6 ± 7.1	4.4 ± 0.6	0	36.6 ± 2.2	11.1 ± 0.8	10.3 ± 4.4
*Clostridium pasteurianum*	DSM 525	50.4 ± 0.2	3.1 ± 0.5	0	75.1 ± 4.3	22.8 ± 2.6	21.0 ± 6.0
**Gram-negative bacteria**							
*Escherichia coli*	DH5α	37.0 ± 5.2	0	0	68.0 ± 6.1	28.0 ± 3.2	0
*Escherichia coli*	ATCC 25922	87.8 ± 3.3	21.0 ± 0.6	0	95.5 ± 3.4	67.8 ± 2.2	6.6 ± 1.5
*Klebsiella pneumoniae*	G31	41.9 ± 1.8	0	0	77.8 ± 2.3	39.1 ± 1.5	16.7 ± 5.0
*Klebsiella pneumoniae*	ATCC 9621	56.2 ± 5.3	0	0	87.6 ± 4.4	35.7 ± 4.2	11.8 ± 2.7
*Pseudomonas mendocina*	ATCC 25411	3.8 ± 0.2	0	0	39.2 ± 1.9	2.4 ± 0.4	0
*Pseudomonas aeruginosa*	NBIMCC 1390	78.1 ± 6.3	12.5 ± 0.3	0	86.8 ± 3.4	66.3 ± 4.3	5.1 ± 2.2
*Pseudomonas stutzeri*	ATCC 50227	54.0 ± 2.0	24.1 ± 3.2	0	93.6 ± 5.0	41.2 ± 3.8	8.2 ± 1.7
*Cupriavidus necator*	NBIMCC 3735	6.4 ± 0.7	0	0	26.3 ± 2.1	13.5 ± 1.7	0
*Alcaligenes faecalis*	ATCC 2072	16.9 ± 2.0	0	0	48.2 ± 3.5	10.5 ± 1.4	0

**Table 3:** Overview of the transcriptomic response of reference strains subjected to 1-butanol and 2-butanol challenge. Only genes upregulated or downregulated >2 times were considered.

DEGs—2-Butanol vs. 1-Butanol FC *	Strain	Total	500–100×	100–50×	50–25×	25–5×
Upregulated genes	*E. coli* ATCC 25922	1039	7	4	22	277
	*B. subtilis* ATCC 168	102	1	2	5	25
Downregulated genes	*E. coli* ATCC 25922	1122	3	6	12	264
	*B. subtilis* ATCC 168	111	0	0	2	31

* FC—fold change; “Total”—number of all DEGs; “×”—fold change in their expression.

**Table 4:** Selected highly regulated genes in *B. subtilis* ATCC 168 under 2-butanol compared to 1-butanol and their counterparts in *E. coli* ATCC 25922. ND = expression not detected, or regulated less than two times.

Gene	Protein	log2 FC * in BS	log2 FC * in EC
*tnpB*	IS200/IS605 family element RNA-guided endonuclease TnpB	7.63	1.32
RS00010	16S ribosomal RNA	6.41	1.60
RS30195	23S ribosomal RNA	6.36	ND
*pyrF*	Orotidine-5′-phosphate decarboxylase	5.35	ND
*carB*	Carbamoyl-phosphate synthase large subunit	5.28	−1.06
*clpC*	ATP-dependent protease ATP-binding subunit ClpC	4.79	ND
*tkt*	Transketolase	4.67	1.92
*pcrA*	DNA helicase PcrA	4.18	ND
RS02965	VWA domain-containing protein	−5.25	ND
*malS*, *maeBs*	Oxaloacetate-decarboxylating malate dehydrogenase	−4.12	1.76
RS01980	YhgE/Pip domain-containing protein	−4.30	ND
*ctaB*	Protoheme IX farnesyltransferase	−4.42	ND

* log2 FC on 2-butanol compared to on 1-butanol. BS—*Bacillus subtilis* ATCC 168; EC—*E. coli* ATCC 25922.

**Table 5:** DEGs encoding transcription regulators.

Strain	Gene	Protein	log2 FC *
**Upregulated**			
*E. coli*	*cspE*	Transcription antiterminator/RNA stability regulator CspE	2.71
	*tdcA*	Transcriptional regulator TdcA	2.61
	*adiY*	DNA-binding transcriptional activator AdiY	2.58
	*caiF*	Carnitine metabolism transcriptional regulator CaiF	2.52
	*clbR*	Colibactin biosynthesis LuxR family transcriptional regulator ClbR	2.40
	RS00140	Cro/CI family transcriptional regulator	3.17
	RS06380	Transcriptional regulator	2.73
	RS17995	CII family transcriptional regulator	2.71
	RS20585	Adhesin biosynthesis transcription regulatory family protein	2.61
	*yjhI*	IclR family transcriptional regulator	2.58
	RS0124285	Helix-turn-helix transcriptional regulator	2.52
	*nikR*	Nickel-responsive transcriptional regulator NikR	2.40
*B. subtilis*	RS20535	Fur family transcriptional regulator	2.70
	RS05340	LacI family DNA-binding transcriptional regulator	2.13
	*phoP*	Two-component system response regulator PhoP	1.68
	*cggR*	GapA transcriptional regulator CggR	1.24
	RS22015	IreB family regulatory phosphoprotein	1.18
	RS20705	MarR family transcriptional regulator	1.13
	*codY*	GTP-sensing pleiotropic transcriptional regulator CodY	1.05
	*ctsR*	Transcriptional regulator CtsR	1.20
**Downregulated**			
*E. coli*	*bglG*	Transcriptional antiterminator BglG	−6.64
	*norR*	Nitric oxide reductase transcriptional regulator NorR	−6.11
	*sgrR*	DNA-binding transcriptional regulator SgrR	−4.13
	*phnF*	Phosphonate metabolism transcriptional regulator PhnF	−3.96
	*ydeO*	Acid stress response transcriptional regulator YdeO	−3.91
	*phoB*	Phosphate response regulator transcription factor PhoB	−3.85
	*mlc*	Sugar metabolism global transcriptional regulator Mlc	−3.69
	*soxS*	Superoxide response transcriptional regulator SoxS	−3.46
	*yfdE*	CoA:oxalate CoA-transferase	−3.37
	RS10895	TetR/AcrR family transcriptional regulator	−3.37
	*lysR*	DNA-binding transcriptional regulator LysR	−3.36
	*hyxR*	LuxR family transcriptional regulator HyxR	−3.33
	RS04225	AlpA family transcriptional regulator	−3.31
	*yhfZ*	GntR family transcriptional regulator YhfZ	−3.31
	*ilvY*	HTH-type transcriptional activator IlvY	−3.29
	*gcvA*	Glycine cleavage system transcriptional regulator GcvA	−3.26
	*ulaR*	HTH-type transcriptional regulator UlaR	−3.13
	*ynfL*	LysR family transcriptional regulator	−3.08
	*lrhA*	Transcriptional regulator LrhA	−3.02
	*punR*	DNA-binding transcriptional activator PunR	−2.85
	RS06610	Helix-turn-helix transcriptional regulator	−2.82
	*dgoR*	D-galactonate utilization transcriptional regulator DgoR	−2.81
	*cysB*	HTH-type transcriptional regulator CysB	−2.80
	*acrR*	Multidrug efflux transporter transcriptional repressor AcrR	−2.64
	*frmR*	Formaldehyde-responsive transcriptional repressor FrmR	−2.63
	*comR*	TetR family copper-responsive transcriptional repressor ComR	−2.60
	RS17080	MarR family transcriptional regulator	−2.57
	RS09695	DeoR/GlpR family DNA-binding transcription regulator	−2.53
	*decR*	DNA-binding transcriptional regulator DecR	−2.49
	*yjhU*	Sugar-binding transcriptional regulator	−2.40
	*dhaR*	Dihydroxyacetone kinase operon transcriptional regulator DhaR	−2.36
	*araC*	Arabinose operon transcriptional regulator AraC	−2.34
*B. subtilis*	*spx*	Transcriptional regulator Spx	−1.49
	RS16600	MerR family transcriptional regulator	−1.86
	RS19530	RsfA family transcriptional regulator	−2.16
	*pyrR*	Bifunctional pyrimidine operon transcriptional regulator	−2.47
	RS02555	YebC/PmpR family DNA-binding transcriptional regulator	−2.94
	RS22070	Rrf2 family transcriptional regulator	−4.06

* FC—fold change on 2-butanol compared to on 1-butanol; *E. coli*—grown at 2% (*v*/*v*); *B. subtilis*—grown at 1% (*v*/*v*).

**Table 6:** Selected highly regulated genes in *B. subtilis* ATCC 168 under 2-butanol compared to under 1-butanol and their counterparts in *E. coli* ATCC 25922. ND = expression not detected, or regulated less than two times.

Gene	Protein	log2 FC * in BS	log2 FC * in EC
*groES*	Co-chaperone GroES	2.40	−1.20
*groEL*	Chaperonin GroEL	2.82	−1.12
*dnaK*	Molecular chaperone DnaK	1.83	−1.79
*spy*	ATP-independent periplasmic protein-refolding chaperone Spy	ND	−4.86
*ibpA*	Small heat shock chaperone IbpA	ND	−4.79
*ibpB*	Small heat shock chaperone IbpB	ND	−4.34
*torD*	Molecular chaperone TorD	ND	−2.90
*dnaJ*	Molecular chaperone DnaJ	ND	−2.69
*hypA*	Hydrogenase maturation nickel metallochaperone HypA	ND	2.17
*cbpM*	Chaperone modulator CbpM	ND	2.03
*hybE*	Hydrogenase-2 assembly chaperone	ND	1.84
*skp*	Protein-export chaperone SecB	ND	1.77
*secB*	Acid-activated periplasmic chaperone HdeB	ND	1.64
*cspD*	Small cold shock-induced protein CspD	−2.56	ND
*cspB*	Small cold shock-induced protein CspB	−3.87	1.53
RS11580	Small cold shock-induced protein	−4.67	ND

* log2 FC on 2-butanol compared to on 1-butanol; BS—*Bacillus subtilis* ATCC 168; EC—*E. coli* ATCC 25922.

## Data Availability

Nucleotide sequences are available in the NCBI GenBank at https://www.ncbi.nlm.nih.gov/sra/PRJNA1178085; https://www.ncbi.nlm.nih.gov/sra/PRJNA1178164 (accessed on 11 November 2024).

## Supplementary Materials

The following supporting information can be downloaded at: https://www.mdpi.com/article/10.3390/ijms252413336/s1.
