# Supplementary material for: Bacterial Tolerance to 1-Butanol and 2-Butanol: Quantitative Assessment and Transcriptomic Response
Source: Int J Mol Sci. 2024 Dec 12;25(24):13336. doi: 10.3390/ijms252413336 (PMC11728337; doi:10.3390/ijms252413336)
Supplement: Supplementary file 1 [file ijms-25-13336-s001.zip › ijms-3310993-supplementary.pdf]

Supplementary material

Bacterial tolerance to 1-Butanol and 2-Butanol: Quantitative Assessment and Transcriptomic Response

Alexander Arsov [1], Penka Petrova [1], Maria Gerginova [1], Lidia Tsigoriyna [2], Nadya Armenova [2], Ina Ignatova [2] and Kaloyan Petrov [2,*]

[1] Institute of Microbiology, Bulgarian Academy of Sciences, 1113 Sofia, Bulgaria;
al.arsov@microbio.bas.bg (A.A.); ppetrova@microbio.bas.bg (P.P.); mariagg@microbio.bas.bg (M.G.);
[2] Institute of Chemical Engineering, Bulgarian Academy of Sciences, 1113 Sofia, Bulgaria;
lidinka29@gmail.com (L.T.); nadq.armenova@gmail.com (N.A.); ina.ignatova@iche.bas.bg (I.I.);
* Correspondence: kkpetrov@iche.bas.bg

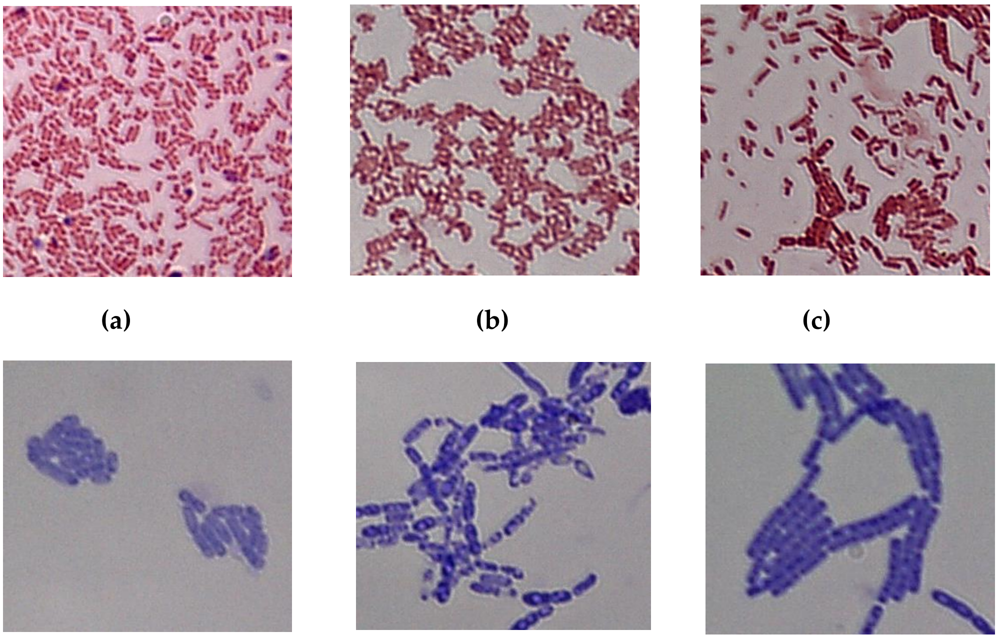

(a) **(b)** **(c)**

(d) **(e)** **(f)**

Figure 1S. The morphological changes of bacteria after 8 hours of 1-butanol and 2-butanol stress. Gram-stained bacteria were presented under the microscopic view by Leica, DMC4500 Digital Microscope Camera, magnification 1000× (Wetzlar, Germany). **(a)** *E. coli* ATCC 25922 in LB; **(b)** *E. coli* ATCC 25922 in LB with 2% (v/v) 1-butanol; **(c)** *E. coli* ATCC 25922, LB with 2% (v/v) 2-butanol; **(d)** *B. subtilis* ATCC 168 in LB; **(e)** *B. subtilis* ATCC 168 in LB with 2% (v/v) 1-butanol; **(f)** *B. subtilis* ATCC 168, LB with 2% (v/v) 2-butanol.
